# Participatory Action Planning to Address the Opioid Crisis in a Rural Virginia Community Using the SEED Method

**Published:** 2020-07-21

**Authors:** Emily B. Zimmerman, Carlin L. Rafie, Dawn E. Moser, Angelina Hargrove, Toni Noe, Courtnaye Adams Mills

**Affiliations:** 1 Center on Society and Health, Virginia Commonwealth University; 2 Human Nutrition, Foods and Exercise, Virginia Polytechnic Institute and State University; 3 Engaging Martinsville, Virginia Polytechnic Institute and State University

**Keywords:** cbpr, seed method, opioids, participatory modeling, action planning, participatory research, stakeholder engagement

## Abstract

The SEED Method is a multi-stakeholder approach that was created to involve diverse stakeholders in the development and prioritization of research questions using community-based participatory research (CBPR) principles. Here we describe an adaptation of the SEED Method that focuses on developing and prioritizing strategies for addressing a health problem and bringing stakeholders together to develop and implement community action plans based on those strategies. We describe steps for implementing the SEED Method for community action planning and the results of a case study in a rural Virginia community with high opioid prescription and mortality rates. A participatory research team worked with three groups of Topic stakeholders to gather data, develop conceptual models, and create and prioritize strategies for reducing prescription and non-prescription opioid misuse and overdoses. Each group came up with 19 to 25 strategies and prioritized their top five, which included actions, services or programs, strategies, policies, and system changes. Attendees at community action planning meetings reviewed the 15 prioritized strategies, proposed three additional strategies, and prioritized their top choices. Community stakeholders started four work groups to implement the selected strategies in collaboration with the research team.

## INTRODUCTION

Community-based participatory research (CBPR) is an approach to creating research in partnerships between communities and researchers (Israel et al., 2012; Wallerstein et al., 2018). An integral part of the process is action to address community-identified needs. Our team implemented the SEED Method in a rural Virginia community to address an issue of great concern to the community: high rates of opioid misuse and overdoses. The SEED Method, developed at the Virginia Commonwealth University Center on Society and Health by Zimmerman and colleagues (Zimmerman, Cook, Haley, et al., 2017), is a mixed-methods approach created to engage stakeholders at multiple levels in the research development process. The term engagement, as used throughout this article, refers to “the meaningful involvement of patients, caregivers, clinicians, and other healthcare stakeholders throughout the entire research process—from planning the study, to conducting the study, and disseminating study results” (Patient-Centered Outcomes Research Institute, 2018), based on principles of community-based participatory research (Israel et al., 2012).

The opioid crisis was a pressing concern in the focus community, and there were ongoing efforts to address it. Community stakeholders, who knew about a previous project completed by our CBPR team using the SEED Method, asked if we could start a project on the opioid issue in their community. Our previous work using this method focused on developing and prioritizing research questions, but we knew that community members wanted to develop strategies and potential solutions. Here we describe an adaptation of the SEED Method that occurs in two phases. Phase I focuses on working with diverse community stakeholders to develop and prioritize potential strategies for addressing the health problem. Phase II brings together stakeholders to develop and implement community action plans based on those strategies. In our case study, the process resulted in a list of potential strategies developed by stakeholder participants. In community action planning meetings, stakeholders selected four of these strategies and formed four work groups for the action phase of planning and implementation.

## THE SEED METHOD FOR COMMUNITY ACTION PLANNING

Patients and stakeholders are increasingly finding opportunities to collaborate throughout the research process (Zimmerman & Concannon, 2021). Engagement, or involvement, allows for the inclusion of their unique experiential understanding and underlying values, and ensures that research priorities reflect their concerns and preferences (Bowling et al., 1993; Kapiriri & Norheim, 2002; Lomas et al., 2003). Collaboration with stakeholders also provides opportunities for improved research outcomes, including validity and relevance (Entwistle et al., 1998).

The action stage of participatory research is an important yet often underdeveloped part of the research process, serving as a bridge between research and next steps for addressing community priorities. Action planning with diverse community stakeholders utilizes local expertise and can leverage organizational resources and bridge silos. Key action planning components include engagement of appropriate individual and organizational stakeholders, community capacity building (Schulz et al., 2011), and creation and prioritization of strategies based on community resources, needs assessments, and priorities. Some action planning examples in the literature include a CBPR initiative to reduce disparities in infant mortality in Florida (Salihu et al., 2011), the Green Communities Canada guide to the School Travel Planning process (Green Communities Canada, 2016; Macridis et al., 2016), and the Healthy Environments Partnerships’ Community Approaches to Cardiovascular Health (HEPCATCH) project to reduce cardiovascular disparities in Detroit, MI (Schulz et al., 2011).

Lack of technical training of stakeholders and a paucity of capacity-building methods can limit the effective engagement of community stakeholders in research (Hoffman et al., 2010; O’Haire et al., 2011; Oliver et al., 2004). Community capacity building involves mobilizing key community stakeholders around a common understanding of the needs and solutions, and providing tools for successful project management. Coalitions of these stakeholders with complementary skills and resources develop group dynamics that create the capacity to act (Zuckerman, 2016). Bringing together the right mix of individual and organizational stakeholders is important to successful implementation of the strategies, and strategic recruitment should be conducted to ensure that cross-sector partnerships develop (Mossberger & Stoker, 2001). Organizations and individuals that have a self-interest in the issue and recognize the need for collaboration to meet their goals will be more likely to dedicate themselves to the effort. Identifying a lead organization that is broadly respected can assist with recruitment of others. Similarly, respected community stakeholders who have roles that span sectors are important to recruiting a broad range of individuals who will engage in the work of implementing strategies to address the community health issue (Mossberger & Stoker, 2001).

The SEED Method was designed in response to a need for evidence-based methods that incorporate best practices, processes, and engagement methods. It can be applied in diverse settings to help stakeholders explore the factors influencing a health issue and prioritize areas for further research and action. Evaluation of two SEED Method demonstration projects found that training in concept modeling and research question development addressed issues of capacity building, as did the facilitation tools created to lead the teams through the process of stakeholder selection, conceptual modeling of the health issue, and research question development and prioritization. Feedback from participants in these projects indicated that they felt well-prepared for the tasks they were asked to perform and that they had a sense of satisfaction in gaining new skills in the process (Zimmerman, Cook, & Price, 2017).

The two phases of the SEED Method for action planning include a strategy development stage and an action planning phase. The highest level of stakeholder involvement in the SEED Method is the participatory **research team**, which represents a collaboration between researchers and community stakeholders. The research team is responsible for project planning and implementation, selection and recruitment of stakeholders, and dissemination of project results. Key steps for the research team are reviewing data, identifying priority stakeholder participants, meeting facilitation, and action planning.

The next level of participation is **Topic groups** composed of stakeholders who are strategically important to the health issue being investigated (e.g., specific subgroups of community members, patients, caregivers, clinicians, policymakers, and service providers). These stakeholders are prioritized by the research team using a structured process, that is, an activity using a specific format (described in detail later). Topic groups participate in major project activities, such as identifying and prioritizing potential community strategies.^1^ They meet over the course of several months, moving through a programmed series of project activities. Key steps for the Topic groups include reviewing data, participatory conceptual modeling, and developing and prioritizing strategies. In the case study project, we involved community members with a history of opioid use or those whose family members had a history of opioid abuse, as well as a diverse set of community members from health care to law enforcement with expertise in how the opioid crisis affects the community. Additional SCAN (Stakeholder ConsultANt) participants are consulted to further diversify stakeholder perspectives. Consultation can take the form of focus groups, interviews, or other short-term involvement. Finally, the action planning phase brings together project participants with a wide range of community stakeholders to finalize strategies and develop work groups to implement them.

Based on CBPR principles, a key ingredient of the SEED Method process is interactivity in meetings and group activities, as well as between the research team, the Topic groups, and the SCAN participants. The key to this process is facilitation that allows for co-learning and shared decision-making, even while completing activities that follow a specified format. Finally, capacity building is an important part of this process. Sharing information, learning new methods, and having the opportunity to try new skills are emphasized throughout (Zimmerman & Cook, 2021).

### CASE STUDY: MARTINSVILLE/HENRY COUNTY AND OPIOIDS

The case study project created local priorities and action planning for the opioid crisis in Martinsville/Henry County, VA (MHC). Martinsville is an independent city surrounded by Henry County located at the foothills of the Blue Ridge Mountains in Southern Virginia and known for outdoor recreation, arts, and cultural events. MHC has a rich history that many do not expect considering the current rates of unemployment, opioid prescriptions, and crime. Once a large farming community and trade center, MHC’s economy shifted from tobacco to manufacturing starting in the early 1900s (Cleal & Herbert, 1970). Furniture, mills, textiles, and other manufacturing expanded through the 1960s (Dorsey, 2017; Gettleman, 2002). By 1980, Martinsville had more millionaires per capita than any city in America (Derks, 2000, p. 246). The prolific days of manufacturing furniture, textiles, mirrors, and nylon diminished as, throughout the 1980s through 2000s, furniture production was outsourced overseas and textile mills closed. Today, MHC is experiencing new economic growth in some key industries (Martinsville Henry County Economic Development Corporation, n.d.).

Nationally, physicians began prescribing new formulations of opioids for chronic pain in the 1990s, and the number of prescriptions, dose, and length of prescriptions increased through 2010. The amount of opioids prescribed in the U.S. was three times higher in 2015 compared to 1999 (Centers for Disease Control and Prevention (CDC), 2017b), though there have been recent decreases. Opioid prescription patterns varied substantially across the country, with six times higher average per capita amounts prescribed in top prescribing counties compared to the lowest prescribing counties. Counties with certain characteristics tended to have higher rates of opioid prescriptions: a larger percentage of non-Hispanic whites, lower educational attainment, higher prevalence of diabetes, arthritis, and disability, higher rates of unemployment and Medicaid enrollment, more dentists and physicians per capita, and higher suicide rates (Guy et al., 2017). Nationally, in 2018, 3.7% of persons 12 years and older reported opioid misuse, with slightly higher rates in males (4%) than females (3.5%). Self-reported misuse was present in all age groups, with highest rates in persons aged 18 – 25 (5.6%) and 35 – 39 (5.1%). The lowest rate of reported misuse was in individuals > 65 years of age at 1.3%. The highest rates of opioid misuse were found among non-Hispanic Native Hawaiians or Pacific Islanders (8.4%), with lowest rates among people of Asian descent (Centers for Disease Control and Prevention (CDC), 2019).

MHC was hit by an opioid crisis, likely exacerbated by the economic turbulence of plant closings and unemployment (Guy et al., 2017). In July 2017, *US News & World Report* reported that Martinsville had the highest per-capita rate of opioid prescriptions. The overall opioid prescription rate in 2016 was of 399.9 per 100,000 residents in Martinsville, compared to 66.5 in the U.S. (Centers for Disease Control and Prevention (CDC), 2017a). Nationwide, the amount of opioids prescribed was the equivalent of 640 milligrams of morphine per person in 2015, compared to more than 4,000 milligrams per person in Martinsville (US News and World Reports, 2017). The three-year average opioid mortality rate in Martinsville was three times higher than the state average for Virginia. The area had the highest rate of emergency room visits involving unintentional opioid overdoses in VA: 32 visits per 100,000 in January 2017, compared to 9.2 visits per 100,000 in VA. That rate rose quickly, from 19.8 per 100,000 three months earlier (Carlton & Collins, 2017). Emergency room visit rates increased for all substances, opioids, and heroin in Martinsville and Henry County in 2018, despite declines in other areas of the state (Virginia Department of Health, 2020). Furthermore, in 2015 the rate of neonatal abstinence syndrome was 19.2 per 1,000 live births, compared with Virginia’s rate of 6.1 per 1,000 live births (Collins, 2018).

Due to the complex factors involved in the opioid crisis, effective intervention requires a multisector response, which in turn requires that a broad array of stakeholders collaborate to identify strategies and implement changes. Experts promote a variety of evidence-based suggestions for intervening in the opioid crisis - from changes in physician prescribing to drug courts. The Institute for Healthcare Improvement argues that community-wide efforts are needed at the national, state, and local levels and emphasizes the need for community stakeholders to collaborate and to create multi-faceted solutions. They list a range of actions (e.g., decreasing supply, improving non-opioid pain management, education, reducing stigma) and actors (e.g., health care providers, justice system, law enforcement, legislators) to help drive change (Martin & Laderman, 2016).

In MHC, various community initiatives were started to address the opioid crisis, including the opioid task force, which consisted of local law enforcement, Piedmont Community Services, New College Institute, SOVAH Health, and non-profit organizations assisting those affected by opioids. Engaging Martinsville, a CBPR team established in 2015 to conduct a demonstration of the SEED Method addressing disparities in lung cancer outcomes, had recently developed a stakeholder research agenda on lung cancer (Rafie et al., 2019). Interest in that project led community stakeholders involved in addressing the opioid crisis to ask Engaging Martinsville to conduct a SEED Method project on opioids. Virginia Polytechnic Institute and State University and Virginia Commonwealth University applied to and received funding from the Corporation for National and Community Service to implement a SEED Method project in Martinsville that would adapt the method by adding a community action planning component.

In the next section we discuss the key steps involved in phase I (stakeholder identification of strategies) and phase II (community action planning) of the method and illustrate implementation and outcomes using the MHC project as a case study. We limit case study details to description of how the SEED Method was implemented. We look forward to publishing the study results and the specifics of working to address the opioid crisis in future publications.

### SEED METHOD PHASE I: STAKEHOLDER IDENTIFICATION OF STRATEGIES

The SEED Method is a multi-stakeholder approach that includes a participatory research team, Topic groups of stakeholders, and SCAN (consulting) stakeholders (see Table 1). We describe this phase in relation to the aims and activities of each level of engagement.

### THE PARTICIPATORY RESEARCH TEAM

Recruiting the participatory research team is one of the first project tasks. Team composition can vary depending on the project. For example, the research team may be composed of laypeople with ties to the target community and/or professionals and service providers who have a strong interest in addressing the health issue. Generally, the team will have one or more researchers or persons familiar with the research methods. Following CBPR principles, the research team shares decision making power and recognizes the different types of expertise that members bring to the project (Wallerstein & Duran, 2006). Although some team members may take the lead on specific tasks because of their expertise and access to resources, it is important that each research team member has the opportunity to participate in all aspects of the project and take on responsibilities in areas that may be new to them. The participatory research team leads the project and has primary responsibility for processes such as selecting and recruiting stakeholder participants, organizing data, and facilitating meetings (see Table 2).

#### KEY STEPS

The research team has goals and tasks throughout the project (Table 3). Here we highlight two key steps: gathering and reviewing data and identifying priority stakeholders for the Topic groups.

Gathering and reviewing data is an early stage of research team work in which all members participate to share the information that they have access to or do research to gather additional information. Examples of types of data to be reviewed and shared by the research team include descriptions of the health issue (causes, prevalence, outcomes, patient demographics, disparities), demographics and history of the geographic area or target population, available services and service gaps, and current and potential policies. The depth of the information should be tailored to the knowledge and expertise of team members. In addition to using published data and program or administrative data, team members can conduct informational interviews. For example, interviews with local health system leaders, service providers and clinicians can provide data on who is at risk in the target population, where people receive services, and what the local challenges are. Alternatively, the team can invite knowledgeable stakeholders to speak at meetings. In addition to providing a shared knowledge base for research team members, the presentations and materials created in this step can be used with the Topic groups and during community action planning meetings and other dissemination opportunities.

The information gathered and shared by the research team is an important step in preparing to identify priority stakeholders. The SEED Method uses a series of *Stakeholder Identification Matrices*^2^ (available in the SEED Method Toolkit)^3^ to facilitate the process of identifying and prioritizing stakeholder groups and recruitment resources. The first matrix prioritizes patient and caregiver subgroups. All research team members participate in this process, which can be led by one or two team members who have familiarized themselves with the SEED matrix and activity guides. Research team members customize the list of subgroups based on the health issue and the target population. Subgroups could include people with specific demographics (e.g., gender, age, race, ethnicity), health status, insurance status, risk factors, and so on. The research team members also choose three selection criteria that are most relevant for their project (e.g., disparities, prevalence, barriers to care). The group then rates each subgroup across the 3 selection factors (low = 1, medium = 2, high = 3) and sums across the selection factors to rank the subgroups. Those subgroups with the highest total score are selected as priority subgroups. Another stakeholder matrix focuses on selecting other stakeholders (such as clinicians, service providers, decision makers), to be customized by the research team.

The research team must decide the total number of Topic groups that will be created (all of our projects so far have created 3 groups), and the number of groups that will be patients and/or caregivers and other stakeholders. The priority subgroups identified in the two selection matrices can be combined to form more heterogeneous Topic groups. The research team then uses the recruitment matrix to plan where and how to recruit Topic group members.

#### CASE STUDY RESULTS

The Engaging Martinsville (EM) research team led the project in Martinsville/Henry County. EM included eight members – two university faculty, two community members who had participated in previous EM projects (one of whom acted as the project coordinator), and four additional community members with personal experience or expertise related to opioid use disorder or treatment. We also had a graduate research assistant. EM held weekly three-hour meetings during phase I of the project, except during the weeks when they were facilitating Topic group meetings.

Research team members prepared and discussed presentations covering basics about opioids, addiction, MHC demographics, and opioid and substance abuse trends in Virginia and MHC. In addition, the EM team had several guest speakers, including representatives from local law enforcement and community mental health services, and the health department. Team members also attended various webinars and other informational sessions and reported information learned to the team.

EM decided to have three Topic groups, one composed of patients and/or caregivers, and two composed of providers and other stakeholders. Using the patient identification matrix, EM identified nine priority subgroups: middle-aged adults, whites, people with injuries or chronic conditions requiring pain management, people with mental/behavioral health problems, people on Medicaid or uninsured, high risk populations, high risk health professionals, spouses/partners of individuals with substance abuse, and people in long-term recovery. The team decided that the patient/caregiver Topic group would consist of patients and family members, with an emphasis on opioid users in recovery, those with a family history or partner with substance abuse disorder, people with mental health or substance abuse disorders, and people with disabilities or chronic conditions. Seven people were recruited into this group.

Using the provider stakeholder identification matrix, seven priority subgroups were selected: emergency medical technicians, emergency department personnel, health care providers, police, social service employees, judicial system employees, and corrections officers. The two Topic groups from the provider stakeholder category were 1) police, judicial, corrections, emergency medical technicians, and emergency room personnel, and 2) clinical providers, recovery center personnel, social services, and counselors. Six and eight people were recruited to these groups, respectively. The team planned logistics for the three Topic groups, including a schedule of evening meetings at the local hospital.

### THE TOPIC GROUPS

The Topic groups collaborate with the research team over the course of several months to explore the health topic and develop strategies that are responsive to community needs, assets, and priorities. Various members of the research team facilitate the Topic group meetings, covering the project overview, data review, focus group and key informant interview planning and review of results, conceptual model training, conceptual modeling, strategy development, and strategy prioritization (see Table 3). These activities require a minimum of about seven meetings, ranging from about 1.5 hours to 3 hours long. The SEED Method Toolkit provides details on the meeting agendas and detailed facilitator guides and scripts for each of the structured activities (i.e., conceptual model training, conceptual modeling, and strategy development and prioritization). Activities are discussion-based, with each stakeholder encouraged to share his or her experience and expertise.

Below we highlight three key steps for the Topic groups: conceptual modeling, creating strategies, and prioritizing strategies.

#### KEY STEPS

Having information relevant to the health topic and its impact on the target community creates critical capacity in stakeholder participants to contribute to the research process. Topics covered and the level of complexity should be tailored to the project and the level of knowledge and expertise of the stakeholder participants. Exchange of information can be bi-directional and include building in opportunities for Topic group participants to present and share data and information. Another form of capacity building is the discussion that occurs naturally within the groups as participants weigh in on the data and share their own experiences and knowledge.

The SEED Method uses participatory conceptual modeling as a core strategy for engaging Topic groups in the process of exploring how contextual, community, system, interpersonal, individual and behavioral factors interact to result in health outcomes of interest. We encourage participants to use a socio-ecological or multilevel framework in considering the potential factors influencing health outcomes and how they are interrelated (see for example Kaplan et al., 2000), though others may choose a systems or other framework to guide the process. The exercise is facilitated by a research team member using guides from the SEED Toolkit. To start the exercise, the facilitator asks the Topic group participants to individually brainstorm all of the factors that might influence the health outcome. After individual brainstorming, participants share and discuss the factors that they identified and each is written on a large sticky note to be used in the modeling session. We generally categorize each factor into a domain (e.g., environment, demographics, social factors) as we discuss it.

**Figure 1.**
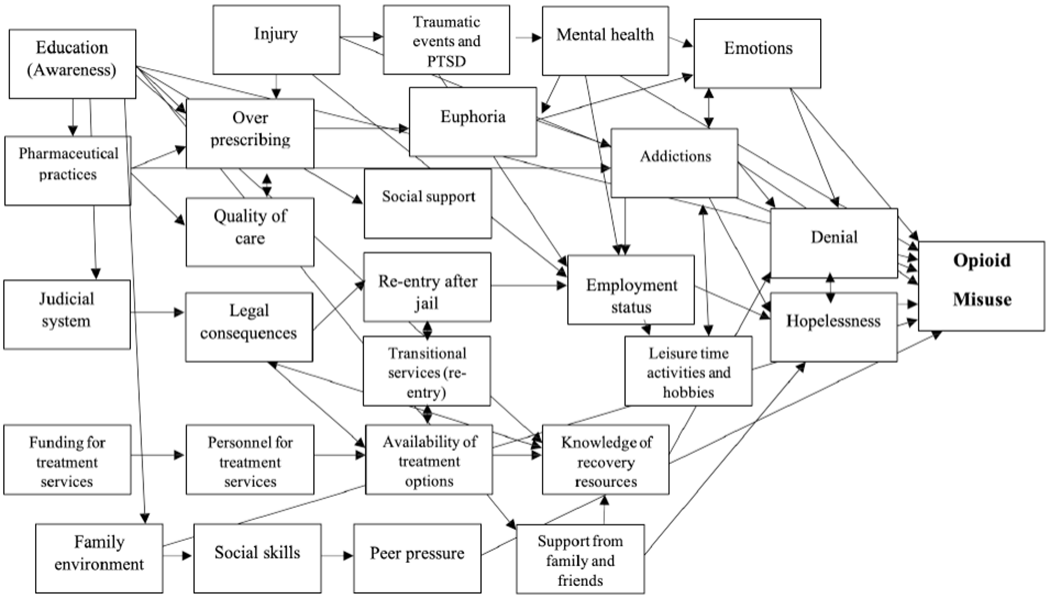
Topic Group Conceptual Model from MHC Study

After participants have discussed all of their factors and categorized them, the facilitator starts the modeling process. We use cause and effect models that diagram the causal sequence of identified factors. The facilitator leads each Topic group through the process of thinking through how the identified factors may be related to the outcome of interest, and the cause and effect sequence that they think makes most sense. We start by defining the health outcome of interest (for example, opioid misuse) and placing that on the far right side of the model. Then, participants take turns selecting the factors and discussing where they should be in the model (what sequence of factors makes sense to them). The sticky notes are convenient for moving factors around as the model evolves. We connect factors that participants think are causally related by arrows (Figure 1). As the facilitator verifies the placement of the arrows on the model it spurs additional discussion of which factors are connected and what might be missing. When the model is finished the facilitator can encourage the group to reflect on where the model points to opportunities for intervention, or how proximity to the outcome may indicate opportunities that might be addressed in the short term (e.g., behavior change) vs. long-term actions that might have potential for greater population impact (e.g., upstream determinants).

Although these causal models have been effective in our projects, other modeling or exploratory techniques could be substituted. Some examples could be systems modeling, ethnographic or narrative inquiry, or arts-based inquiry. The point is to engage in an exploratory process that allows participants to think in a systematic way about important factors related to the health outcome and how these might inform strategies to address it.

**Figure 2.**
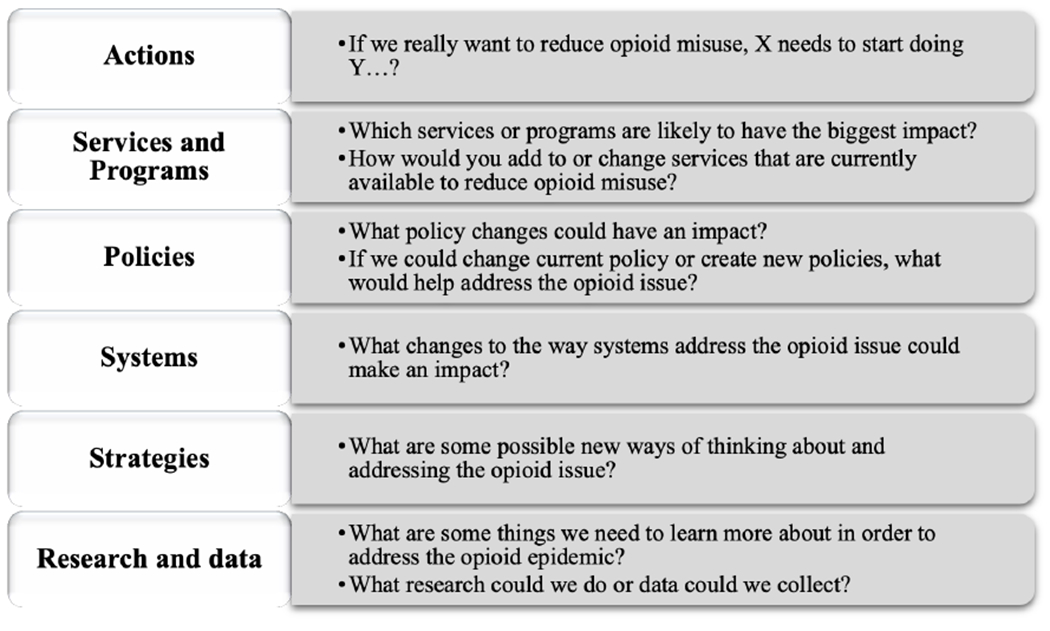
Prompts Used to Facilitate Strategy Development

After creating and reviewing their models, each Topic group meets to develop strategies. Strategies could be new or revised programs, policies, research, systems, or other interventions. As with the previous activity, this session is facilitated by a research team member using SEED toolkit guides. The facilitator starts the session by reviewing the conceptual model the group created and the conceptual models created by other groups. This can spur a useful conversation among Topic group members about how the models differ across Topic groups. The facilitator then provides a series of prompts (customized to the project) that encourage participants to develop a range of different types of strategies (see example in Figure 2). Participants generally write down as many strategies as they can in response to each prompt. When the prompts are finished, they choose which ideas to share with the group. Each strategy is discussed by the group.

In the next meeting, each Topic group reviews the list of strategies created by its members. The creator of each strategy is asked to remind the group what the strategy is about and why he/she thought it was important. Group discussions may lead to revising some strategies or combining similar strategies. Then the group votes on their top strategies, focusing on what is important but also feasible. Generally, the research team decides in advance the target number of strategies to prioritize in each group. For example, a Topic group that has created 25 strategies may end up prioritizing their top five.

#### CASE STUDY

As shown in Table 3, the MHC Topic groups met seven times to participate in data review, focus group planning, review of focus group findings, conceptual model training, conceptual modeling, strategy development, and strategy prioritization. Topic group members were also invited to participate in Phase II.

During the conceptual modeling exercise each Topic group selected the outcome of interest and developed its own model. The Community group selected ‘entering treatment and/or recovery’ as the outcome and developed a model that focused on physical and mental health, service availability and quality, family relationships, attitudes, and spirituality. The Service group selected ‘opioid misuse’ as the outcome and created a model that focused on physical and mental health, treatment options and knowledge, the justice system and re-entry, the family environment, and attitudes (see Figure 1). The Health Providers group selected ‘prevalence of opioid use disorder’ as the outcome. That group created a model focused on trauma and mental health, community and social factors, family and work, and availability of treatment.

Each group was asked to create strategies to address their outcomes of interest. We used the prompts shown in Figure 2 to facilitate the task. For each prompt we provided further explanation and examples. Each group came up with 19-25 strategies and prioritized their top five, which included actions, services or programs, strategies, policies, and system changes.

**Figure 3.**
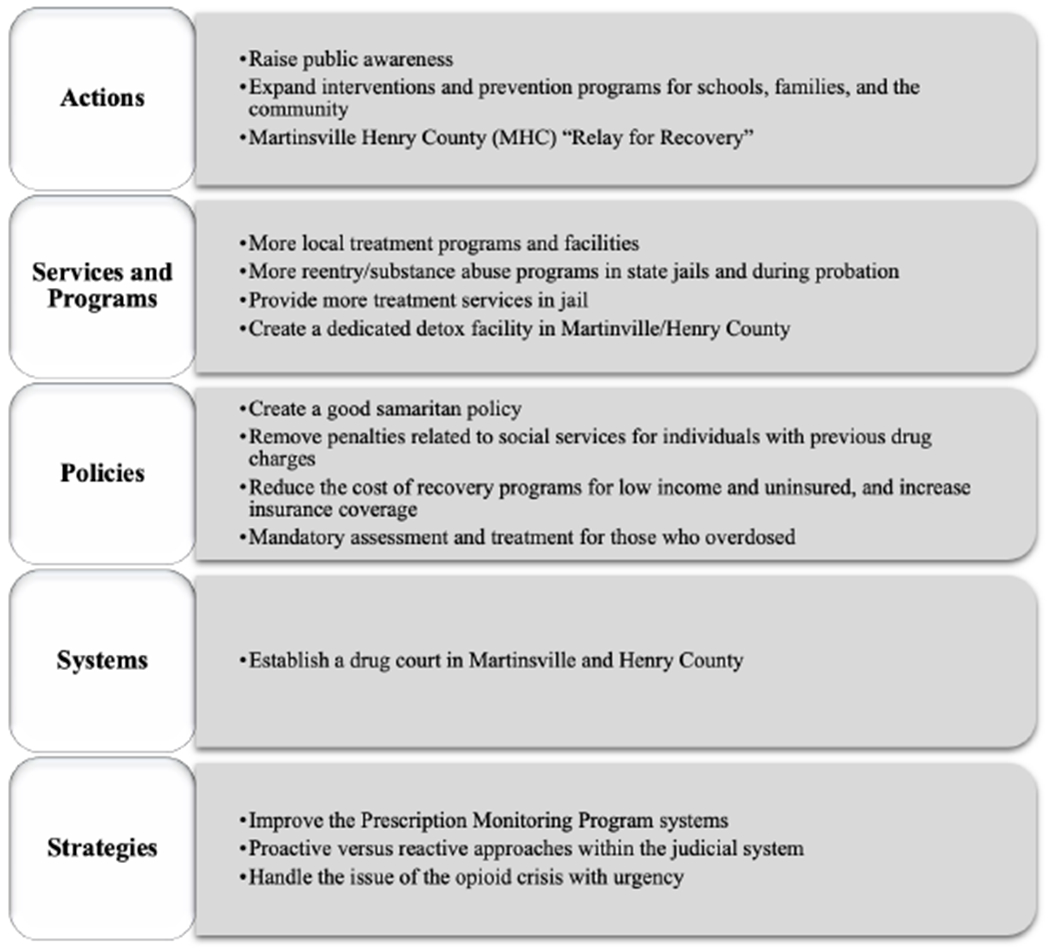
Strategies Prioritized by Topic Groups in MHC to Address the Opioid Crisis

In the following meeting, each Topic group reviewed and discussed the strategies that its members had created and voted on their top priorities. The list of prioritized strategies is shown in Figure 3.

After the final Topic group meeting, participants were invited to take part in Phase II (action planning).

### THE SCAN PARTICIPANTS

The SEED Method includes consulting SCAN particpants to gain additional perspectives from stakeholders not represented in the Topic groups. The intentions of this step are two-fold (1) to provide a contextual background and greater understanding of the experiences of diverse stakeholders, and (2) to broadly explore relevant risk factors. Projects select a consultative method to gather data, such as focus groups, interviews, or storytelling. The data are collected and summarized by research team members and discussed by the research team and the Topic groups. The findings are intended to inform the conceptual models and strategies created by the Topic groups, therefore this step should be completed before the Topic groups develop their conceptual models.

#### KEY STEPS

Key steps in gathering information from SCAN participants include identifying stakeholder groups, developing recruitment strategies, recruiting stakeholders, developing questions, planning and conducting data collection according to the method selected, summarizing data, and holding discussions about the findings.

#### CASE STUDY

EM and the Topic groups worked collaboratively to identify SCAN participants. During the second Topic group meeting, we asked participants to discuss which stakeholders they would like to recruit for focus groups. As they discussed their ideas, a research team member listed them on flip chart sheets and helped organize them before facilitating a multi-voting process. Three focus groups were identified by the Topic groups, and one by the EM Team. Selected focus groups included 1) family and friends of opioid users (selected by the Treatment Provider group), 2) people providing recovery services (selected by the Community group), 3) people providing treatment services (selected by the Service Provider group), and 4) decision/policy-makers (selected by EM).

Topic group and research team members developed specific questions for each stakeholder group related to their specific experience. The questions were generated to explore pathways to opioid misuse, barriers to treatment, stigma, community awareness, policies, prevention, and programs. With guidance and help from the academic researchers on the EM team, an initial draft of the questions and prompts was formulated, which was then discussed and refined by the whole team.

Community residents on the EM team then took the lead on recruitment, identifying organizations in the community and calling on existing contacts. Similar to the recruitment of the Topic groups, the EM research team identified potential locations for recruiting focus group participants and used email, multi-media, and direct communication to find participants. Five to seven participants were in each group, for 24 total participants.

Community members on the EM Team who expressed an interest were chosen to moderate the focus groups. Two academic team members provided training to the EM members for approximately 6 hours, across two meetings. To increase the comfort level of the members who would serve as focus group moderators, we held mock focus groups. Each 90-minute focus group took place in conference rooms in a local hospital. EM team members obtained informed consent.

**Figure 4.**
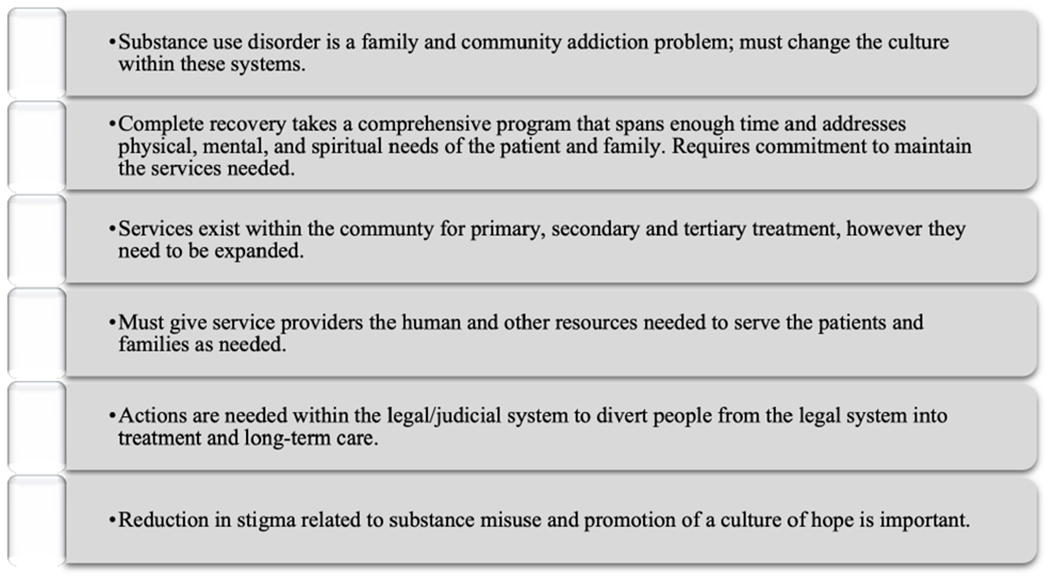
Sample of themes found in MHC focus groups

A content analysis of the focus groups was conducted by academic members of the EM team. A synopsis of the findings was presented to each Topic group by EM team members. The information from the SCAN participants provided additional background and perspectives on the opioid crisis in the Martinsville community. Multiple themes emerged from the focus groups, including the impact on families, feelings of helplessness, and lack of a drug court and help within the judicial system, among others. A list of some common themes from each focus group are included in Figure 4.

### SEED METHOD PHASE II: COMMUNITY ACTION PLANNING

Creating a final list of priority strategies identified by community stakeholders in the first phase of the SEED Method is an important first step to addressing the community health issue. Mobilizing the community to action on these strategies is the next step, and is essential for the work of phase I to have an impact. The activities of phase II include community prioritization of strategies in community-wide action planning meetings, forming work groups composed of key community stakeholders, and supporting the activities of the work groups through capacity building activities and technical assistance.

### COMMUNITY PRIORITIZATION OF STRATEGIES

#### KEY STEPS

Two community-wide action planning meetings are conducted with the goals of introducing the final list of priority strategies to relevant community members and organizations, selecting strategies for immediate action, and forming work groups of dedicated community actors to develop and implement action plans for each strategy. To recruit a wide range of community stakeholders, the community research team identifies key organizations and individuals in the community who are relevant to the priority strategies. In addition, all Topic group and focus group participants are invited. Multiple outreach strategies are utilized (e.g., email invitations, personal invitation, print and social media). In preparation for the first meeting, the community research team prepares background information for each strategy. The information should include the intended outcomes of the strategy, resources needed, assets in existence in the community, and a brief summary of the evidence related to the strategy. A concise presentation is prepared for the meeting with information about each strategy.

The objectives of the first action planning meeting are to provide an overview of the SEED process that generated the priority strategies, present information about each strategy, and select strategies that the community stakeholders will work on in the coming year. In order to recognize the expertise and priorities of stakeholders who were not involved in Phase I, attendees are invited to propose a limited number of additional strategies. Additional strategies that receive broad support from the rest of the meeting participants are added to the list of strategies for voting. Meeting attendees select priority strategies through a multi-voting or other consensus process. The number of priority strategies chosen will depend on the resources available to the community research team for supporting implementation. At the conclusion of the meeting, the final priority strategies are reviewed and participants are invited to the second action planning meeting.

The objectives of the second action planning meeting are to form a work group of committed community members for each strategy, outline the timeline of work group activities for the coming 12 months, and begin development of a logic model for each strategy. The meeting should begin with a review by the research team of the strategies that were selected at the first action planning meeting. The work group objectives and timeline of activities are discussed and attendees are asked to join a work group. Each team reviews the roles and responsibilities of work group members and the community research team liaison. A brief training on how to create a logic model is provided, and work groups spend the rest of the meeting beginning work on the logic model for their strategy. Prior to leaving the meeting, the team selects a work group chair and establishes the schedule and location for their meetings.

#### CASE STUDY

The EM team held an action planning meeting with 34 local stakeholders. After reviewing the fifteen prioritized strategies selected by the Topic groups, attendees at the action planning meeting proposed three additional strategies. These received broad support from the group and were added to the list prior to the voting process. We limited the number of strategies that could be selected for action in the coming year to three. This number was chosen based on the human resources available on the research team to support the work, and attendance at the action planning meeting. The final three strategies selected for action in the coming year after completion of the voting process were:

- Establishing a drug court in Martinsville/Henry County
- Creating a dedicated detox facility in Martinsville/Henry County
- Raise public awareness of everything the community is doing to address the issue, what else needs to be done, and how bad the issue is.

Two additional strategies were recommended by attendees at the meeting, and received sufficient support to be included:

- Expand youth and parent substance misuse prevention programs in the schools in Martinsville and Henry County.
- Increase the number of “sober residences” for long-term recovery.

We encouraged meeting participants to come to the second action planning meeting and to invite additional individuals who would be key to successful accomplishment of the chosen strategies. Results from a satisfaction survey completed by attendees showed strong satisfaction with the meeting, with over 90% of attendees rating it is “Excellent”.

The EM team held the second action planning meeting four weeks later, with 21 community members attending. The five final strategies were reviewed and attendees selected the strategy that they would work on in the coming year. There were not enough people willing to commit to the work of increasing the number of sober residences for long-term recovery, so we did not form a work group for this strategy. An EM member was assigned to each work group. Subsequently, the EM team provided a brief training on how to develop a logic model and the work groups began initial steps in developing their logic models. Prior to ending their discussion, work groups scheduled the date, time, and place for their monthly meetings.

##### STRATEGY WORK GROUPS

The work groups are central to the accomplishment of the prioritized strategies. The research team and community participants work together to create a support structure and build capacity to implement each strategy and maintain the engagement of work group members. A research team member is designated as a liaison for each work group and is responsible for team communications, meeting scheduling and reminders, and documentation. Each work group designates a Chair, who is responsible for managing the meetings, creating agendas, and monitoring timely progress of group activities. Work groups schedule meetings at least monthly for the duration of the project period. Contact between work groups and the research team includes a quarterly meeting with work group chairs to exchange information and identify any technical assistance needs of the work groups and semi-annual meetings that bring together all work group members.

#### KEY STEPS

We hope that having work groups organized around preselected strategies will create momentum and counteract some of the challenges faced by multi-sector collaborative initiatives, such as differing goals or understanding of the problem, contributors’ narrow channels of influence, and flexibly sharing resources and responsibilities (Fawcett et al., 2010). Key steps for each work group include developing a logic model (McNamara, n.d.; Renger & Titcomb, 2002), a detailed work plan, and a timeline to guide implementation and work toward a sustainability plan, if needed, at the end of that time.

#### CASE STUDY

The project work groups have been meeting for about 6 months so far. They are in different stages of implementing their work plans, including identifying additional stakeholders, funding opportunities, policy support and useful tools. For example, the work group on establishing a detox center has received funding for additional technical assistance and the prevention group is identifying a curriculum that can be implemented across all schools in the community. Each work group will be able to receive a small grant from project funds to help with planned activities.

### RESOURCE AND TIME CONSIDERATIONS

We often receive inquiries about the cost of using the SEED Method. This is a difficult question to answer because the method was designed to be scalable. Variables such as the number of research team members and how they are paid, the number of Topic groups and participants, and the number of focus groups, interviews, or other consulting participants will all affect the total cost. In addition, some projects may use only some portion of the SEED Method. In order to help readers assess potential costs, Table 4 presents cost estimates for projects of varying scope as well as the costs related to the case study presented in this article. The case study budget excludes indirect or overhead costs that went to other units in the participating universities and is based largely on funded rather than final costs. As the Table illustrates, it is possible to implement a small scale project with minimal funds if personnel time is either volunteer or covered by employers. Larger scale projects and those relying on soft funds (e.g., outside grants) to cover personnel costs will require greater financial resources.

Human resources are the primary requirement for a successful SEED project, and represent the major financial costs associated with it. Members of the research team dedicate the most time and effort throughout the project. We have found it beneficial to have at least one member of the research team who is skilled in various research methods; experience with participatory research is also strongly recommended. A research team member with good organizational skills who can act as project coordinator is an essential component to success. At least one member of the research team should be able to serve as a community liaison, with connections to a range of stakeholders. When the research team is being created specifically for the project, adding members with experience related to the health topic is a good approach.

The timeline for a SEED project is somewhat flexible. Nine months to a year is a good starting point for each phase if implementing all project activities. Key activities that impact the time required to complete the project include recruiting research team members, research team orientation and skill building, Topic group selection and recruitment, Topic group meetings, focus group and interview data collection, and community action planning meetings.

### LESSONS LEARNED

Each participatory research project has the potential to add to collective learning about what works. The SEED Method presents some specific challenges because it is a fairly structured set of specific activities that can take place over several months or years. Within the structure provided by the method, team members and stakeholder participants must have a say in project decisions. Within that longer time frame it can be a challenge to keep everyone involved, especially when family, work, and personal commitments arise.

**Lesson 1:** Your team should decide together how to implement the SEED Method. How often will you meet? Who will take the lead on certain activities? How should the activities be customized to the setting and participants? For example, if voting is not a good fit for prioritization, what is? If focus groups are not a good fit for your stakeholders, what would work better? How many Topic groups can the team work with?

**Lesson 2:** Keep revisiting who is at the table and how to encourage them to stay involved. Our team experienced a lot of turnover in the action planning phase. After a full year of working together, some had to move on to fulfill other commitments, so we sought out others who could take their place. We did not budget stipends for our work group Chairs, but given the importance of their role we think offering them a stipend is a great idea.

**Lesson 3**: Find ways to recognize and incorporate community expertise throughout the project. This strengthens the knowledge base, promotes community buy-in, and helps keep the project connected to the larger community. Some examples in the case study included developing the project proposal with community stakeholders, asking community agencies to suggest research team members, bringing in representatives of key community agencies to present to the research team about how opioids have affected the community and how they are addressing the problem, and inviting community members to participate throughout the action planning process.

### STRENGTHS AND LIMITATIONS

One frequent response we have gotten to the SEED Method is that it is different from what community stakeholders expected or have engaged in before. Many community residents become involved in issues that are of great concern to their communities, but sometimes the groups and coalitions that form to address community concerns lack a systematic process for moving forward from discussion to action. By providing a series of steps that engage stakeholders to learn about the issue and learn from each other, create and share ideas, and develop implementation plans, the SEED Method offers a tool that can be used for a range of projects, from research development to planning new services and interventions. That work takes time, but it should be time well spent.

We sometimes get questions about handling conflict. We have seen limited conflict related specifically to using SEED. In a previous project where we encountered conflict on the research team we invested extra time in explaining the project timeline and activities and creating project ‘roadmaps’ to help everyone understand where we were in the process. During the case study project, there were some differences of opinion in the community about the value and appropriateness of particular strategies for addressing the opioid crisis. We encouraged all participants to put forward their ideas for strategies, and the two levels of prioritization meant that only those priorities with the most support moved forward. In this way, no ideas were excluded but controversial and less popular ideas were not prioritized. As the ‘community perspective’ below helps to illustrate, a structured project can help ease some of the tension that comes from tackling a big issue like the opioid crisis.

## Community Perspective: “You are only responsible for the effort, not the outcome”

In 2017, the CDC reported that patients in our city were prescribed more opioids than anywhere in the United States. In 2018, our community had one of the highest opioid related deaths in Virginia. In 2019, I found myself in the ICU, holding the hand of a mother whose daughter died from an unintentional overdose. There were heavy feelings of hopelessness and urgency. How would conducting focus groups and developing conceptual models result in saving lives? Today?

I joined this project determined to make the effort, free of bias, and accept the outcome. The SEED Method, with clear objectives and principles, was a reassuring road map. Our role was to review evidence and generate strategies to engage a diverse range of community stakeholders. We were not to be experts on opioids, rather, curious observers and interpreters.

In addition to the stigma associated with substance use disorders, scientific studies and evidence-based programs were sometimes misunderstood in our rural community. Our principal investigators and project coordinator were models of calm neutrality. I admired their Socratic approach which inspired critical thinking and discussions, challenging me to broaden my skill set and collaborate in new ways. I valued the direction and critique to focus less on minute details and more on the collective progress and dynamic process.

Today, in our final year of the project, the makings of remarkable actions are taking place as we work toward viable and sustainable plans, policy, and funding. Seeking to listen and understand, I discovered that each of us, in spite of our differences, are investing our best efforts toward positive change, which remains a truly gratifying and unexpected outcome.

— Courtnaye Adams Mills, Engaging Martinsville Research Team Member

An immense advantage of collaborating with community stakeholders is that each brings a unique perspective. Their knowledge, passion and eagerness cannot be replicated, especially those who have been personally affected (see ‘Community Perspective’ below). The Engaging Martinsville research team was formed with community stakeholders who shared their expertise and knowledge in a previous SEED Method project addressing lung cancer mortality disparities in MHC (Rafie et al., 2019). Two of these participants enthusiastically agreed to continue with Engaging Martinsville and assisted with recruitment of other community members for the opioid project. Four new community members were added to the Engaging Martinsville team, and brought knowledge, family, work and personal experience. The team of six community members, along with two experienced researchers, and a doctoral student, collaboratively began working together as Engaging Martinsville. The community research team members recruited Topic group and SCAN participants with influence in the community along with lay individuals with a personal experiences to share, eagerness to learn new skills and try something new, investment in their community, and a desire to have a voice in addressing health priorities.

## Community Perspective

“Being a part of the Engaging Martinsville team is an incredibly rewarding experience. Community stakeholder involvement takes a multifaceted approach to attack the opioid crisis head on. Meeting with community members from all walks of life provides different ideas, opinions and insights into the issues that directly affect the community. I have had the privilege of participating in the Engaging Martinsville group from the beginning. Focus groups and Topic groups provided knowledge and points of views that have helped me not only see the opioid crisis differently, but also the community in a different way. As a community, coming together to fight the battle and not only come up with ideas but to further turn those ideas into actions provides hope to the community in which we live.

Opioids have impacted me personally and had a devastating effect on my life. I lost both of my parents to opioid overdoses. Since that time, opioids have somewhat defined my life. I joined the Engaging Martinsville team for this project with the passion to contribute to my community and in the hopes to help prevent others from experiencing what I have. I watched both of my parents struggle with addiction from a young age and saw firsthand the effect on them and my family. Losing my mother as a young adult, I could see the gaps in care as she struggled time and again to get clean. There weren’t many options other than a brief stint in the hospital and then a referral to an already overrun community agency with no follow up. This allows those struggling to fall through the cracks. Even the ones that want to get the help they need have limited options in the community. Additionally, there is no detox center that can aid those in need and help them detox from substances safely and with the needed counseling and medical care. Being a part of this group has sparked community conversation into providing services that are needed in our community. I feel that bringing the community together for these conversations has set the wheels in motion to create and carry out the action plans needed to help our community in a positive way.”

— Toni Noe, Engaging Martinsville Research Team Member

Although team dynamics for Engaging Martinsville were encouraging, collective, and productive, we experienced some of the typical challenges of participatory research. For example, the research team members were hired to work part time, and all but one had a full-time day job, limiting their availability. A team member who did not work days was helpful in dispersing documents and recruiting Topic group members and SCAN participants. When scheduling events and meetings, some Topic group members and SCAN participants were available to meet during the day, but the Engaging Martinsville team was unable to accommodate those times due to work schedule conflicts. As documented in countless other study reports, successfully engaging the community requires flexibility, humility, and respect.

There are many ways to engage stakeholders in research and community action. Having a process that is documented and tested is useful for planning as well as for communicating with funders and stakeholders. As creators and experienced users of the SEED Method, we relied heavily on the tools and guidance in the SEED Toolkit, but also found ourselves making many decisions tailored to the project, the community, and the health issue along the way. We foresee changes and improvements as the process is used and hope that it will continue to evolve with input from users. Given the variability in goals and resources for participatory research, we feel it is important that the SEED method is easily adaptable to address any health issue and is versatile enough to be used in different contexts.

## Figures and Tables

**Table 1.** Overview of SEED Method Process for Community Action Planning

**Phase I: Identify Strategies**	
**Identify and engage** (12 - 20 weeks)	**Research team:** Meets weekly or bi-weekly, gathers and reviews data on the target population and health issue, completes the SEED stakeholder identification and recruitment matrices, recruits TG participants, holds first TG meetings. **Topic groups:** First TG meeting to get introduced to the project.
**Consult** (4-6 weeks)	**Research team:** Facilitates TG meetings, conducts focus groups and interviews and summarizes results. **Topic groups:** Each TG meets twice to plan focus groups and interviews and to review results. **SCAN participants:** Participate in focus groups and interviews (or other consultative methods).
**Conceptualize** (2 weeks)	**Research team:** Facilitates TG meetings, reviews conceptual models. **Topic groups:** Meet twice to participate in conceptual model training and then to create conceptual models.
**Generate strategies** (1-2 weeks)	**Research team:** Facilitates TG meetings. **Topic groups:** Each TG meets to review the full set of models and participates in a facilitated exercise to generate strategies.
**Prioritize strategies** (1-2 weeks)	**Research team:** Facilitates TG meetings. **Topic groups:** Each TG meets to prioritize strategies.
**Phase 2: Action Planning**	
**Select strategies** (2-4 weeks)	**Research team:** Researches each prioritized strategy, prepares a presentation for community stakeholders, and holds a community stakeholder meeting to select final strategies. **Topic groups:** Although TG work is finished, TG participants can attend the community stakeholder meeting and stay involved through the action planning phase.
**Form work groups** (2-4 weeks)	**Research team:** Holds second community stakeholder meeting to review final strategies, form work groups, and select work group chairs. **Work groups:** Each WG develops a meeting schedule and a logic model.
**Implement work plans** (10 months)	**Research team:** Research team liaisons attend WG meetings, provide support and documentation, and report back to the research team. The team tracks progress, provides technical support, and holds a semi-annual meeting that brings all WG participants and other community stakeholders together to review progress and address challenges. **Work groups:** Meet regularly, create a work plan and timeline, and implement work plan steps. Chairs meet with research team quarterly.
**Wrap up/next steps** (4 weeks)	**Research team:** Holds a meeting that brings all WG participants and other community stakeholders together to review progress, celebrate achievements, and plan for next steps. **Work groups:** Develop a sustainability plan (as needed).

Note: TG = Topic groups, WG = work groups

**Table 2.** Participatory Research Team Roles and Responsibilities

**Phase I**	
Gather and review data	May include existing data, informational interviews, guest speakers, local services and policies
Complete SEED Stakeholder Identification Matrices	Identify priority stakeholders and resources for recruitment of Topic groups
Topic group selection and recruitment	Select number and types of stakeholder Topic groups
Topic group planning and logistics	Develop a meeting schedule (time and place) for each Topic group; prepare materials (e.g., data, description of method, schedule, contact sheets)
Focus group and interview (or other consultative method) planning and training	Research team members provide and participate in training on conducting focus groups and interviews; facilitate interview question development; recruit focus group and interview participants; plan and conduct meetings
Summarize focus group data	Discuss findings and summarize data to share with Topic group participants and other stakeholders
Facilitate Topic group meetings	Facilitate and document all Topic group meetings
Review and finalize deliverables	Review and edit conceptual models and lists of strategies
Research and present on final strategies	Do background research on the priority strategies selected by the Topic groups; prepare a presentation for the action planning meeting
**Phase II**	
Hold action planning meetings with community stakeholders	Identify and invite stakeholders; set up meeting logistics; conduct meetings
Prepare materials for work groups	Assemble needed information and materials for work groups
Support work groups	Liaison with work groups; document work; provide technical assistance; review progress

**Table 3.** Monthly overview of activities – Phase I

Month	Research team	Topic groups	SCAN participants
1	Recruit and onboard research team	NA	NA
2	Kickoff meeting, overview of SEED Method, review data, guest speakers, human subjects certification, project logistics, social media	NA	NA
3	Review and summarize information, select Topic groups using *Stakeholder Identification Matrices*	NA	NA
4	Topic group logistics and recruitment	NA	NA
5	Topic group logistics and recruitment, Topic group meetings, focus group and interview training	Meeting 1 (kickoff, project overview)	NA
6	Topic group meetings, focus group and interview planning and recruitment	Meeting 2 (review of data, focus group planning)	Focus groups
7	Focus groups, summarize focus group and interview results, Topic group meetings	Meeting 3 (review of focus group/interview data), Meeting 4 (conceptual modeling training), Meeting 5 (conceptual modeling)	NA
8	Topic group meetings, review of conceptual models, logistics for action planning meetings	Meeting 6 (strategy development)	NA
9	Topic group meeting, review of prioritized strategies	Meeting 7 (strategy prioritization)	NA
10	Research on prioritized strategies, recruit and plan for action planning meeting	NA	NA

## Footnotes

1 In previous projects, the Topic groups focused on developing and prioritizing research questions.

2 The Toolkit contains the following matrices to aid in stakeholder selection: *Patient Stakeholder Identification* (for patients and caregivers), *Service Provider/Other Stakeholder Identification*, and *Stakeholder Recruitment*

3
https://societyhealth.vcu.edu/work/the-projects/the-seed-method-for-stakeholder-engagement.html
